# Human-Computer Interactive English Learning From the Perspective of Social Cognition in the Age of Intelligence

**DOI:** 10.3389/fpsyg.2022.888543

**Published:** 2022-04-06

**Authors:** Qilin Yan

**Affiliations:** College of Foreign Language, Jiangxi University of Technology, Nanchang, China

**Keywords:** intelligent age, social cognition, human-computer interaction, English teaching, universal language model

## Abstract

Under the wave of globalization, the ties between countries are getting closer and closer. Based on the differences in the languages of different countries, the importance of English as a universal language is becoming more and more prominent. In the past, English teaching was mainly taught by teachers and students. This mode of English learning is more of theoretical teaching, which has little effect on improving English ability. In the era of intelligence, with the upgrading of technology and the renewal of ideas, interactive English teaching is more accepted by people and becomes a new solution for English teaching. Studying English teaching from the perspective of social cognition, this paper proposes to realize human-computer interaction with the help of advanced science and technology, create an interactive teaching environment, and realize interactive learning in an English environment. This paper designed a control experiment of the experimental group and the control group to verify the impact of human-computer interaction teaching on students’ English performance and investigated students’ performance and teachers’ feelings and their related opinions on human-computer interaction learning. The experimental results of this paper show that human-computer interactive English teaching can help to enhance students’ interest in English learning and improve English expression significantly.

## Introduction

At present, globalization has become a development trend, and cross-border transactions are becoming more and more frequent. Based on this, as a universal language, English is playing an increasingly important role. The best way to learn a language is to communicate freely in a native language environment, so the effect of language learning should also be judged by the degree of communication. Unfortunately, although the importance of English has been emphasized, Chinese students have been learning English in elementary school, junior high school, high school, and even university and postgraduate levels, Chinese students’ English communication skills have not been significantly improved.

The long-standing phenomenon of Chinese English learners is that their listening, speaking, and writing abilities are very uneven. Many students do not have many difficulties in reading, writing, and listening, but they do have many problems in speaking. Most students cannot speak English openly, in other words, our English teaching is silent English. This phenomenon, on the one hand, exposes the biggest problem in the teaching of spoken English, and on the other hand points out the direction of improving English proficiency. As the main place for English learning, the classroom should play a greater role. In the course, the teacher is an English communicator and the student is an English learner. Good interaction between the two can enhance both sides’ understanding of English and further promote English communication. But for a long time in the past, our English teaching only stayed in the one-way transmission stage, the teacher was only responsible for teaching, the students were only responsible for listening, and there was very little interaction. The essence of classroom English teaching should be the interaction between teachers and students, but how to achieve high-quality language interaction within an efficient time frame becomes the focus of our future research.

This paper is based on human-machine interactive English learning from the perspective of social cognition. It emphasizes that English teaching should abandon traditional written teaching and create a good interactive learning environment to help students to better understand, recognize, and master English. Its purpose is to achieve multiple breakthroughs in English listening, speaking, reading, and writing, better help students to grow, improve teachers’ English teaching ability, and improve students’ English performance.

Based on the perspective of social cognition, this paper has the following advantages: (1) introduce human-computer interaction technology and create a good English learning environment with the help of modern equipment to help students to learn English better; and (2) introduce the theory of social cognition and use the theory to better guide students to learn a second language and master English listening, speaking, reading, and writing skills; (3) emphasize that English teaching should abandon the traditional written teaching, create a good interactive learning environment, and realize the interaction between teachers and students, between students, and between students and learning resources, by investigating the effects on students. This helps students to better understand, recognize and master English, improve teachers’ English teaching ability, and improve students’ English performance.

## Related Work

For English interactive teaching, domestic and foreign experts and scholars have achieved certain results. Cole reviews published research on the use of interactive reading aloud in the teaching of English Language Learners (ELLs). He emphasizes the practical application of research findings to help classroom teachers and other educators to make instructional decisions that promote effective and equitable instruction. He uses qualitative content analysis methods to identify topics. To investigate the teaching of English in three different categories: pedagogy, language, and culture. Although many aspects of effective interactive reading are similar for ELLs and mainstream students, this article focuses on interactive reading elements that are different or particularly important to ELLs (Cole et al., 2017). Parvin conducts research on distance education, leveraging new technologies to increase access to the forefront of education and training opportunities. Information and Communication Technology (ICT) includes all technologies used for information processing and communication. He wants to identify a suitable technology to develop a virtual interactive teacher training program for disadvantaged English teachers. He selects respondents through random sampling and analyzes the data using descriptive statistics and quantitative themes. Based on the input of secondary English teachers, he determines their level of access and acceptance of ICT and conducts a needs analysis. He hopes that the research can train disadvantaged rural English teachers (Parvin, 2017). Zhao proposes a three-stage reading teaching method from the aspects of cognitive mechanism and communicative teaching method. Ninety non-English majoring college freshmen participated in the study, who took academic English reading courses in their second semester. Data are obtained from two self-reported research skills assessments and semi-structured interviews, with paired samples t-test and Pearson’s correlation analysis. The results shows that: (1) “Critical thinking,” “Organizing thinking” and “Seeking information” were considered to be significantly improved at the end of the course; (2) reading ability correlates with writing ability and a sense of the “big picture” in self-perception; and (3) cognitive-based interactive reading teaching is recognized as beneficial to academic reading (Gao et al., 2017). Yang designed an interactive teaching optimization (ITLO) algorithm for optimizing the control gain of a proportional-integral (PI) control loop for a voltage source converter-based HVDC system. He achieves broader exploration by introducing multiple classes in a Teaching-Based Optimization (TLBO) algorithm, and then, utilizes Small-World Networks (SWN) for deep interactive learning among teachers or students in different classes to achieve more accurate use. As a result, ITLO is able to effectively avoid local optima due to a proper trade-off between exploration and exploitation (Yang et al., 2019). Tan presents a variety of 1D Finite Difference Time Domain (M1-D FDTD) methods for mobile interactive teaching and learning of wave propagation in transmission lines (TL). Using the M1-D FDTD method, multiple TLs, stubs, and circuit elements can be efficiently modeled. They are easily implemented on mobile devices and applied to mobile interactive teaching and learning of TL topics. These themes are clearly illuminated through interactive visualizations on mobile devices. Using the unconditionally stable M1-D FADI-FDTD method, it is possible to “fast forward” the simulation and increase efficiency by using a time step larger than the stability constraint. Student surveys and tests demonstrate the effectiveness of the method (Tan and Heh, 2019). Huang studies the experimentality and effectiveness of various technology-assisted formative assessment (TAFA) tools, focusing on their pedagogical advantages in assisting the teaching of specific language skills (reading, writing, spelling, etc.). He studies the various affordances perceived by a group of English as a Foreign Language (EFL) teachers in China using a learning management system “designed for the purpose of formative assessment (FA).” He collects data from interviews, teacher journals, and EFL classrooms. The results show that the platform provides EFL teachers with a wide range of pedagogical, managerial, assessment, social, and developmental affordances, while FA experienced teachers report qualitatively and quantitatively better affordances (Huang et al., 2021). Graves explores three English Language Teaching (ELT) curriculum approaches that are currently influential in teaching English in public schools in primary and secondary schools, and how the theory of each approach translates into curriculum practice. These approaches are Communicative Language Teaching (CLT), Genre-Based Pedagogy, and Content and Language Integrated Learning (CLIL). To maintain consistency between methods, he briefly describes the theoretical underpinnings of each method in terms of a matrix of curriculum factors, followed by a discussion of the research on how each method has been implemented in primary and secondary school settings, the extent to which the theory has been put into practice, and the implications for its factors for success in the classroom. And, he discusses the implications for the future of ELT curriculum development (Graves and Garton, 2017). Padmadewi investigates appropriate strategies for teaching English to students with Autism Spectrum Disorder (ASD) in the general classroom. The research has been conducted in the form of a case study of a bilingual school in North Bali, with data collected through observation and interviews. The findings suggest that Visual Media Individual Education Programs (IEPs) offered through co-teaching, differentiated teaching, and “partnership programs” have been found to be suitable for helping students to learn English as a foreign language (Padmadewi and Artini, 2017).

## Social Cognitive Theory and Speech Signal Processing Methods

### Social Cognitive Theory

**Figure 1:**
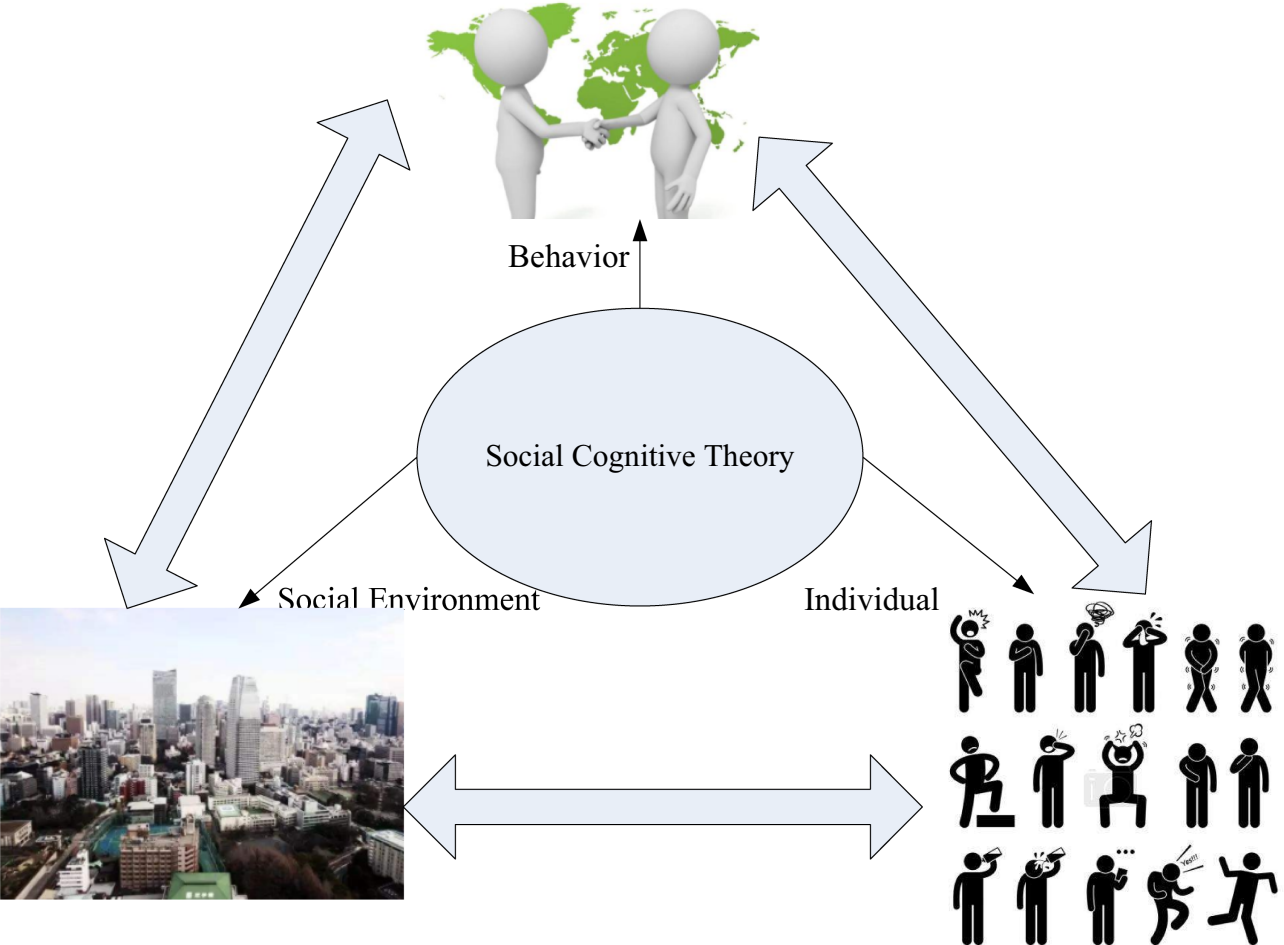
Structure diagram of social cognition theory.

Social cognitive theory believes that there is a ternary interaction between individuals, environments and behaviors, as shown in Figure 1, that is, individuals, environments and behaviors are causal and interrelated (Stewart et al., 2020). According to this theory, individual behavior changes with individual and environmental factors. At the same time, the theory emphasizes the subjective initiative of individuals, and emphasizes that individual factors are an important concept in social cognitive theory, which are manifested in self-efficacy and outcome expectations (Farmer et al., 2018). Self-efficacy is a variable of individual differences in a given situation, and it represents beliefs about the ability to be successful in a given task. Outcome expectations are personal predictions of the outcome of their actions. Environmental factors include physical environment and social environment (Rozado et al., 2017). The physical environment is the surrounding environment in which an individual finds himself; the social environment mainly includes the social relationships and cultural environment in which people can interact and communicate; and behavior is the way people act or respond to specific things or situations (Mencarini et al., 2019).

Social cognitive theory has three mechanisms (Abbas et al., 2019): (1) the decisive role of the individual in the behavior, the individual’s emotions, attitudes, and knowledge will guide the behavior, and the results of the behavior will affect the individual’s emotions and attitudes; (2) environmental factors affect the intensity of individual behaviors, and individuals can also exert their subjective initiative by changing the environment to adapt to human needs; and (3) the function of the individual is the product of the influence of the environment, but the objective environment can also be changed by the individual’s cognition, which mainly depends on the individual’s cognition degree.

Social cognitivist approaches to language teaching take an interactionist stance emphasizing the importance of the interactive and social dimensions in second language learning (hereafter abbreviated as L2). It has been argued that Second Language Acquisition (hereafter abbreviated as SLA) occurs when the language learner’s internal mechanisms engage in linguistic and social contexts, which also occurs when second language learners engage in social interactions. Since L2 is not to obtain it, but to solve problems and complete tasks, it must be combined with other social cognitivist components such as activities, people, and objects. Furthermore, social cognitivist approaches argue that L2 teaching is a valuable component of SLA and should not be separated from learning. A social cognitivist approach to L2 teaching suggests that L2 learners are not viewed as passive recipients of knowledge or as isolated acquirers of L2. Instead, L2 learners are seen as active participants in the teaching process and as true performers of what comes naturally. This suggests that in social contexts, including at school or at home, second language learners are entrenched in such social contexts to become competent participants in culturally, socially, and politically shaped environments. The social cognitivist model of second language teaching incorporates the core principles of second language teaching: the psychological cognitivist imperative and the sociocultural imperative of the second language classroom pedagogy. All of these principles help to shape the teaching of language knowledge combined with language skills. The social cognitivist approach aims to “cultivate communicative learners, but also 21st century multicultural lifelong learners who can participate and contribute” to promote social transformation in a global society. These principles are a synthesis of the research-based insights of social cognitivists, and they embody the basic principles of teaching L2 teachers in actual classroom practice. As is mentioned earlier, it is important to note that these principles are not meant to be followed in a strict order, nor are they mutually exclusive. Taken together, they provide a useful foundation for facilitating classroom instruction for effective L2 learning for students, specifically (Roussou et al., 2018):

1. L2 is effective learning through social interaction and collaboration and between learners and teachers. Teaching is seen more as a process of dialog and interaction, where L2 knowledge and skills are constructed synergistically, rather than the delivery of discrete units of linguistic knowledge in monolo.
2. Language teaching and learning requires the integration of student-centered and teacher-centered activities. Pedagogical approaches seek more innovative naturalistic, experiential, or contingent learning processes, rather than prioritizing formal learning processes, teaching and intentional learning in classroom practice.
3. Language teaching and learning should aim to develop the communicative competence, fluency and linguistic accuracy of language learners. This principle reflects that the recommended pedagogy is more aimed at curriculum innovation to achieve communicative fluency, especially at the discourse level, rather than at achieving L2 formal accuracy.
4. Language teaching and learning well-being involves a balanced emphasis on all aspects of communicative competence components, with due regard to pragmatic authority. Teaching objectives are more aimed at developing and accessing students’ abilities for the learning and use of a second language, rather than focusing on pre-specified teaching and assessing language goals.
5. Language teaching and learning involves a balanced focus on language knowledge (grammar and vocabulary) and language skills (listening, speaking, reading and writing) explicitly and contingently. This principle is in line with curriculum innovation, where the syllabus highlights language standards and language skills rather than being organized according to language standards alone.
6. Paying attention to form is a necessary feature of the second language classroom teaching method. Among them, L2 teaching should clearly attract students’ attention to the classroom.
7. Language teaching requires a combination of inductive and deductive classroom teaching. This principle is consistent with the principle of curriculum innovation in China. All recommended classroom activities should allow students to discover the language rules themselves and involve the explicit representation of language.
8. Assessment requires traditional and alternative methods of assessment using contextualized self, peer and teacher methods.
9. Language instruction requires consideration of individual learners’ social realities and psycholinguistic abilities by adopting differentiation and making content and tasks relevant to their differences.
10.  ICT needs to be integrated into society to develop a twenty-first century-focused teaching process for multilingual L2 learners using an integrated Computer Aided Language Learning (CALL).
11.  Language teaching and learning requires the integration of language and culture in order for learners to become competent cross-cultural speakers who understand the language and behavior of different cultures and establish themselves as speakers of another language.

### Principle of Speech Signal Processing

**Figure 2:**
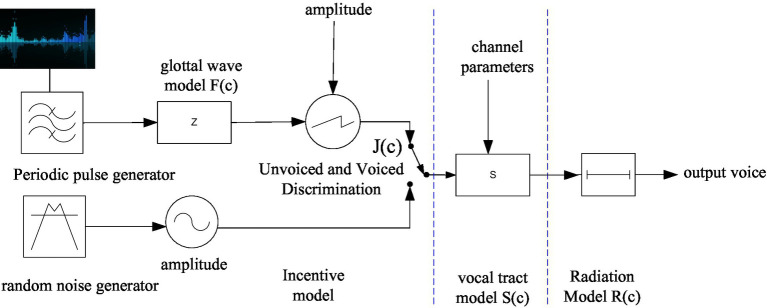
Digital model of speech signal generation.

The speech generation system is divided into three parts: gate system, channel system and radiation system. The “glottic subsystem” is responsible for generating excitation and is located in the lower part of the glottis; the “acoustic channel system” is the channel connecting the glottis and the lips, responsible for signal transmission; the “radiation system” is the site of sound, which is on the outside of the lips (Peng, 2022). Figure 2 is a mathematical model of speech signal generation.

The mathematical model of the speech signal is closely related to the excitation model, the vocal tract model and the radiation model, and its transfer function *F*(*c*) can be expressed as:


(1) F(c)=J(c)S(c)T(c)

where *J*(*c*) represents the excitation signal, and *J*(*c*) is obtained from periodic excitation pulses *via* the glottal waveform *B*(*c*) and gain control in the case of muddy sound. In the case of clean sound, *J*(*c*) is obtained from random noise through gain control. *D_s_* is the amplitude factor of the unit burst, and k is a constant.


(2) $$J\left(c\right)=M\left(c\right)B\left(c\right)=\begin{array}{c} D_{s}\\ 1-c^{-1} \end{array}\cdot \begin{array}{c} 1\\ {\left(1-e^{-kT}c^{-1}\right)}^{2} \end{array}$$

*S*(*c*) represents the channel transfer function, generally using the all-pole model.


(3) $$S\left(c\right)=\begin{array}{c} 1\\ 1-\overset{N}{\underset{h=1}{\sum }}g_{h}c^{-h} \end{array}$$

where N is the model order and *g_h_* is the model coefficient.

The radiation model T(c) is related to the mouth shape and can usually be expressed as:


(4) $$T\left(c\right)=\left(1-tc^{-1}\right),t\approx 1$$

The key to speech signal preprocessing is to enhance the high-frequency part of speech, which includes operations such as speech acquisition, preprocessing, frame segmentation and windowing (Schiavo et al., 2019). In general, a first-order digital filter is a commonly used method and can be expressed as:


(5) $$F\left(c\right)=1-\gamma c^{-1}\left(\gamma \to 1\right)$$

The most commonly used technique for adding windows is the overlapping and overlapping window technique, and the rectangular window and the Hamming window are the more commonly used window functions. The main lobe of the rectangular window is narrow and the frequency resolution is higher, but the side lobes are also higher. Therefore, the interference is serious, and energy leakage is prone to occur. At the same power, the Hamming window has a smaller signal amplitude and smaller side lobes than a rectangular window, so there is less energy leakage. In this paper, the Hamming window is selected to add a window, and the whole process can be expressed as:


(6) $$p_{q}\left(i\right)=p\left(i\right)\cdot q\left(i\right)$$

Endpoint detection is another operation that processes speech signals and is a technique used to determine the location and information content of speech signals. Endpoint detection extracts the part with voice activity and ignores the silent part, which saves a lot of computation and reduces the extra computational overhead of speech signal processing. It examines parameters based on the time-frequency domain and statistical characteristics of speech. Commonly used parameters are short-term energy and average short-term zero-crossing rate, spectral entropy and complexity (Bennett et al., 2018).

The principle of the parameter test method of short-term energy and average short-term zero-crossing rate is to distinguish the voiced and unvoiced sounds of speech signals. Generally speaking, voiced sounds have higher energy in the short term and unvoiced sounds have lower energy. We can construct the short-term energy function, and distinguish the turbid according to the difference of the short-term energy. Let Z be the frame length, *Lz* is the short-term energy *x_z_*(*l*) of frame z, and the calculation formula is as follows:


(7) $$L_{z}=\overset{Z-1}{\underset{l=0}{\sum }}x_{z}^{2}\left(l\right)$$

The short-term energy can distinguish the clear and voiced, but the voice signal is complex, and the degree of discrimination for the turbid energy signal is not high. Therefore, the concept of average short-term zero-crossing rate is proposed. Clear speech is mostly high frequency, showing a high zero-crossing rate, while turbid speech is mostly low-frequency and has a low zero-crossing rate. By definition, the average short-term zero-crossing rate can be expressed as:


(8) $$W_{z}=\begin{array}{c} 1\\ 2 \end{array}\overset{Z-1}{\underset{l=0}{\sum }}\left|\mathrm{sgn}\left[x_{z}\left(l\right)\right]-\mathrm{sgn}\left[x_{z}\left(l-1\right)\right]\right|$$

In the formula, sgn[*x*] is the sign function, that is


(9) $$\mathrm{sgn}\left[x\right]=\{\begin{array}{c} 1,\left(x\geq 0\right)\\ -1,\left(x<0\right) \end{array}$$

**Figure 3:**
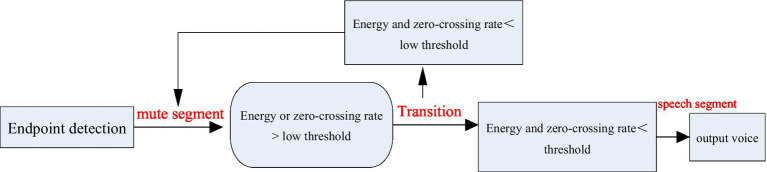
Two-level judgment method for voice endpoints.

According to the above properties, the combination of the two can better discriminate language endpoints, as shown in Figure 3.

Another parameter of endpoint detection is the frequency entropy parameter test. When the language signal is in different states, the entropy value varies greatly. Under noise, the entropy value is large; under silence, the entropy value is small. The frequency entropy parameter test is based on this principle. The basic process is as follows: fast Fourier Transform can efficiently and quickly use computer to calculate discrete Fourier transform and reduce the number of discrete Fourier transform multiplications. Obtain the spectrum of the signal by means of fast Fourier transform (FFT), determine the energy distribution *R*(*v*) at the frequency point v, and calculate the probability density of the spectral components at the frequency point v in the total amount, which can be expressed as:


(10) $$P_{x}=\frac{R\left(x\right)}{\sum_{v=0}^{N-1}R\left(v\right)},x=1,2,\ldots ,N$$

Among them, the number of FFT transform points is N. Since most of the speech signal energy is concentrated between 200 and 3,500 hz, for the convenience of calculation, let the frequency components other than the set energy be 0, namely:


(11) $$R\left(v\right)=0,v\leq \left[\begin{array}{c} 200\\ y_{r} \end{array}*N\right]orv\geq \left[\begin{array}{c} 3500\\ y_{r} \end{array}\cdot N\right]$$

Among them, *y_r_* is the sampling frequency, [*U*] represents the largest integer less than U, and [*U*] represents the smallest integer greater than U. The spectral entropy of each frame is defined as follows:


(12) $$F=-\overset{N-1}{\underset{v=0}{\sum }}P_{v}\cdot \log P_{v}$$

Thus, the onset of speech can be determined by spectral entropy.

The last parameter of endpoint detection is *A*_0_ complexity. In general, complex actions can be split into regular actions and random actions. *A*_0_ complexity is used to describe random actions. Assuming that there is a complex motion time series *c*(*t*), the parameter detection steps are as follows:

Perform discrete Fourier transform on a frame of signal *c*(*n*), there are:


(13) C(v)=G[c(n)]

1. The average value P of the amplitude spectrum can be obtained:

(14) $$P=\begin{array}{c} 1\\ N \end{array}\overset{N-1}{\underset{v=0}{\sum }}C\left(v\right),1<<v<N$$

V is the frequency domain variable and N is the length of *C*(*v*). Taking the average value of the frequency components as the limit, the part larger than the average value is a regular contribution, and the part smaller than the average value is not a random contribution.


(15) $$C^{\prime }\left(v\right)=\{\begin{array}{c} C\left(v\right),ifC\left(v\right)>C\\ 0,ifC\left(v\right)\leq C \end{array}$$

2. Perform inverse Fourier transform *F*^−1^(·) on the spectrum *C*(*v*) contributed by the regular part, that is, *c*_1_(*n*), the regular part-time series.

(16) $$c_{1}\left(n\right)=F^{-1}\left[C^{\prime }\left(v\right)\right]$$

3. Use the following formula to find the complexity *A*_0_ of the frame signal.

(17) $$D_{0}=\overset{N-1}{\underset{n=0}{\sum }}\left|c_{1}\left(n\right)\right|$$

(18) $$D_{1}=\overset{N-1}{\underset{n=0}{\sum }}\left|c\left(n\right)-c_{1}\left(n\right)\right|$$

(19) $$A_{0}=\begin{array}{c} D_{1}\\ D_{0} \end{array}$$

**Figure 4:**
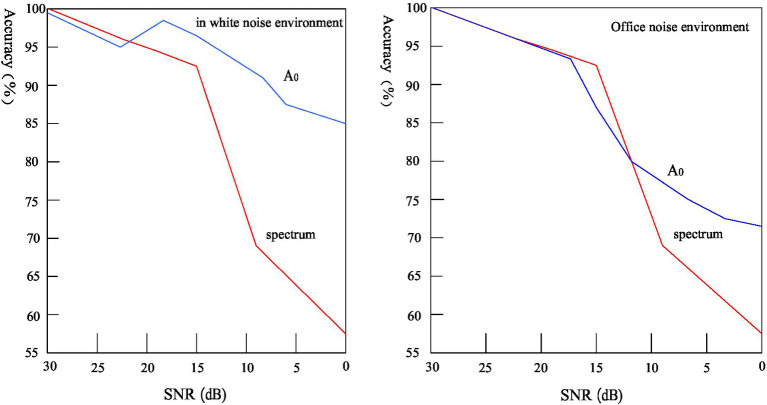
Comparison of voice endpoint detection effects.

Signal-to-noise ratio, the English name is called SNR or S/N (SIGNAL-NOISE RATIO), also known as signal-to-noise ratio, refers to the ratio of signal-to-noise in an electronic device or electronic system. The parameter detection method based on short-time energy and average short-time zero-crossing rate does not work well under the condition of low signal-to-noise ratio, but the endpoint detection algorithm based on spectral entropy and *A*_0_ complexity has a relatively high accuracy (Bagherniya et al., 2017). Figure 4 shows the comparison of endpoint detection accuracy of spectral entropy and *A*_0_ complexity under different signal-to-noise ratios.

Spectral entropy and *A*_0_ complexity parameter detection methods are suitable for parameter detection in low signal-to-noise ratio environments. However, when the signal-to-noise ratio is reduced to a certain level, under the conditions of stable and unstable office noise, the *A*_0_ complexity detection result is better than the spectral entropy, and the accuracy is higher. Therefore, the parameter detection method based on *A*_0_ complexity can effectively overcome the influence of the sound environment on the speech parameter detection system, and adapt to the requirements of a robust speech recognition system.

Linear predictive analysis is a method of predicting current and future data with the help of past data, and its premise is that the data are correlated (Flores-Vázquez et al., 2020). The minimum residual prediction error is a commonly used method for calculating linear prediction coefficients. The minimum residual method is a way for judging whether the actual point is an abnormal point. We can predict the difference between the results and the real results of predicting the development and change of the object according to the minimum residual method. The prediction coefficients represent the characteristics of speech signals and can be expressed as:


(20) $$\hat{r}\left(m\right)=\overset{k}{\underset{x=1}{\sum }}\beta_{x}r\left(m-x\right)$$

where k is the prediction order and *β_x_* is the linear prediction coefficient. The prediction residual *δ*(*m*) is:


(21) $$\delta \left(m\right)=r\left(m\right)-\hat{r}\left(m\right)=r\left(m\right)-\overset{k}{\underset{x=1}{\sum }}\beta_{x}r\left(m-x\right)$$

Define the transfer function *D*(*c*) between the output residual function *δ*(*m*) and the input function *g*(*m*) as:


(22) $$D\left(c\right)=1-\overset{k}{\underset{x=1}{\sum }}\beta_{x}c^{-x}$$

To minimize the squared value of the prediction residual, then:


(23) $$\delta =E\left[\delta^{2}\left(m\right)\right]$$

(24) $$\begin{array}{c} \partial \left[\delta^{2}\left(m\right)\right]\\ \partial \beta_{x} \end{array}=0,\left(1\leq x\leq k\right)$$

Let Φ(p,q)=E[r(m−p),r(m−q)] , then:


(25) $$\delta_{\min}=\Phi \left(0,0\right)-\overset{k}{\underset{x=0}{\sum }}\beta_{x}\Phi \left(0,x\right)$$

### Human-Computer Interaction

**Figure 5:**
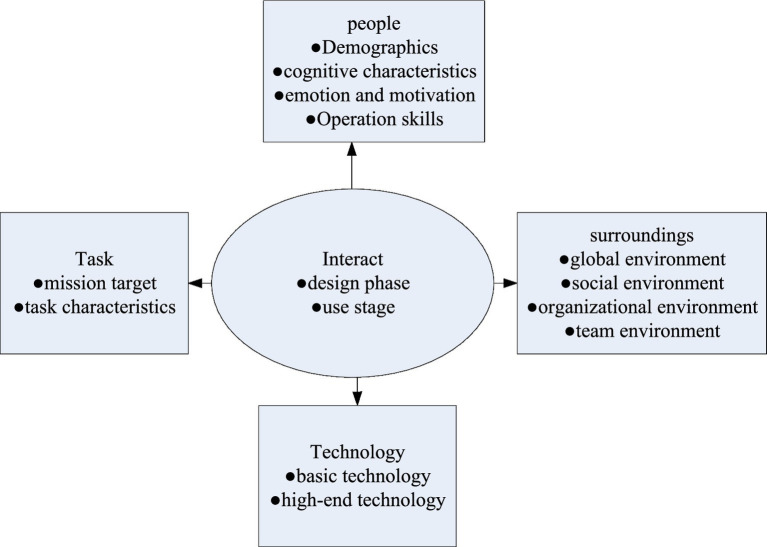
Components of human-computer interaction and their relationships.

Human-computer interaction is the interaction between people and information, technology, and tasks in a certain business, management, organization, and cultural environment (Yeo and Quigley, 2018). As shown in Figure 5, human-computer interaction consists of five factors: people and technology are the two basic factors. The former involves demographic characteristics, cognitive characteristics, emotion and motivation, and operational skills; the latter involves basic technology (such as input and output devices) and high-end technology (such as personalized interface functions). The interaction between people and technology is the core factor, which involves related issues in the stage of technology design and use. Task and context are two auxiliary factors. The former includes the goals and characteristics of the task, while the latter includes the global environment, social environment, organizational environment and team environment.

In English teaching, human-computer interaction is better to use machines to help achieve further interaction between teachers and students, such as live teaching, online correction and other methods are a good application of human-computer interaction teaching. With the gradual integration of various information elements into classroom teaching, it provides students with rich auditory and visual information by constructing real scenes, and provides strong support for students’ language acquisition. In the context of interactive teaching, multimedia has changed from a traditional teacher’s cramming teaching tool to a source of students’ cognition under constructivist interaction. The rapid development of informatization makes multimedia gradually become an extremely important auxiliary tool in classroom teaching. The rational application of multimedia has a very large role in promoting the acquisition of learners: first, students collect and interpret various information involved in the course in advance by using the Internet. After that, in classroom learning, teachers can make full use of more and more diversified multimedia technologies to construct teaching scenarios, and use audio, pictures, videos and other methods to vividly explain the content of the course to students. Finally, students use multimedia to review and deeply understand the key points of the course, so as to complete their construction of the content they have learned.

**Figure 6:**
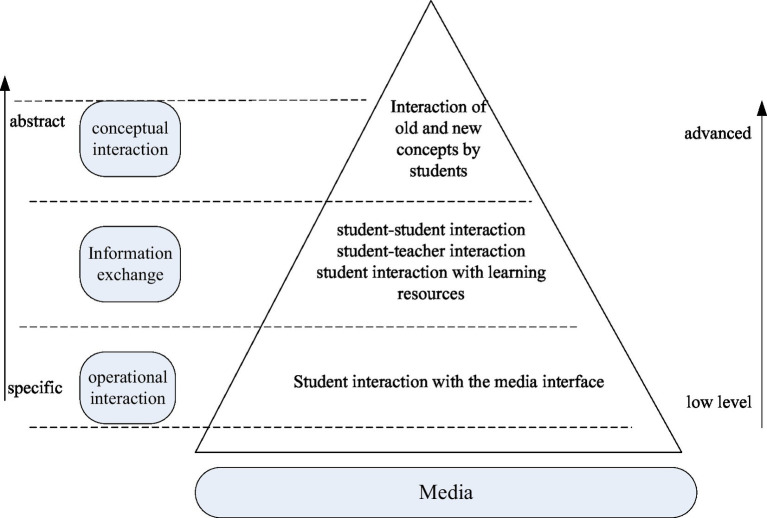
Teaching Interaction Hierarchy Tower.

The goal of interaction in network teaching is two-way interaction to complete the learning task. The interaction level in online education can be divided into operational interaction, information interaction and conceptual interaction, that is, the hierarchical structure of interaction, as shown in Figure 6 (He, 2011). Operational interaction is the interaction of the media interface with the student’s ability to use the media. Information interaction is the embodiment of teaching design and teaching implementation. Conceptual interaction is a key point in evaluating whether instructional interaction promotes effective learning, and this interaction hierarchy is based on interactive media. Learners interact with media to reach teachers and learners, and learners interact with information. Finally, the construction of meaning (conceptual interaction) is accomplished through various interactions. Conceptual interaction is the beginning and end of teaching interaction, focusing on learners’ conceptual interactions is the key to understanding whether interactions can achieve effective learning. The goal of pedagogical interaction is to promote the interaction between old and new concepts through the effective interaction of information (Lazem and Dray, 2018).

## Human-machine Interactive English Learning Experiment and Analysis

### Experiment Content

In order to better test the application of human-computer interaction teaching to English teaching, we selected two classes to conduct experiments to verify the impact of human-computer interaction on teacher-student teaching. The research lasted for one semester. There were about 40 students in each class. The average age of the students was 16 years old. The overall level of the students in the two classes was similar. The experimental group was taught by human-computer interaction, while the control group was taught in the traditional way. Before and after the experiment, a test was conducted on the students’ performance, and a questionnaire survey was conducted on the students’ motivation and interest in learning English to test the results of human-computer interaction teaching.

### Experimental Subjects

Students from two classes were selected to carry out experiments to verify the impact of human-computer interaction teaching on the improvement of English ability. The personnel situation of the two classes is shown in Table 1.

**Table 1:** Introduction to the basic situation of the two classes.

	Experimental class	Control class
Number of people	40	40
Number of male	19	20
Number of female	21	20
The average score (fraction)	71	72

In order to ensure the effect of the experiment, we designed a course plan for human-computer interaction teaching, as shown in Table 2.

**Table 2:** Schedule of experimental courses.

Time	Experimental class (human-computer interaction teaching)	Control class
Monday	Appreciation of reading aloud in English	Traditional teaching methods in class
Tuesday	English Corner	
Wednesday	English Movie Appreciation	
Thursday	English listening game + grammar explanation	
Friday	In-class test	

At the same time, the results of the questionnaire were tested, as shown in Table 3.

**Table 3:** Questionnaire reliability and validity test.

Category		Project	Result
Trust level analysis		Cronbach’s *α* coefficient	0.723
Validity analysis	Kmo	0.761	
	Bartlett’s sphericity test	Approximate chi-square	321.83
		Df	1.249
		Sig.	0.0001

As can be seen from Table 3, the questionnaire is reasonable. Reliability is the stability or reliability of the measurement results, which refers to the degree to which the same object is repeatedly measured by the same method, and the results obtained are consistent with the previous measurement results. Validity is accuracy and authenticity, and refers to the degree to which a measurement tool or means can accurately measure what needs to be measured. Reliability is the premise and foundation of validity, and validity is the purpose and destination of reliability. Any measurement will be scientific only if the dialectical unity of the two is achieved. Cronbach’s alpha reliability coefficient is greater than 0.6, KMO value is greater than 0.6, and Bartlett’s sphericity test sig. <0.05, indicating that the requirements of the table meet the validity and reliability test, and it is a qualified questionnaire.

### Data Analysis

**Figure 7:**
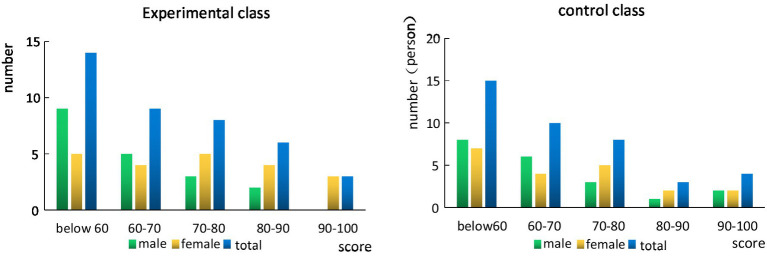
Comparison of the scores of the experimental class and the control class before the experiment.

As can be seen from Figure 7, although there are a lot of people in the high segment of the control class and the experimental class, the proportion of people in the low segment is relatively large, resulting in a low average grade of the class. Overall, women’s English scores are better than men’s. The score distribution of the experimental class is relatively uniform, while the score distribution of the control class has a certain gap.

**Figure 8:**
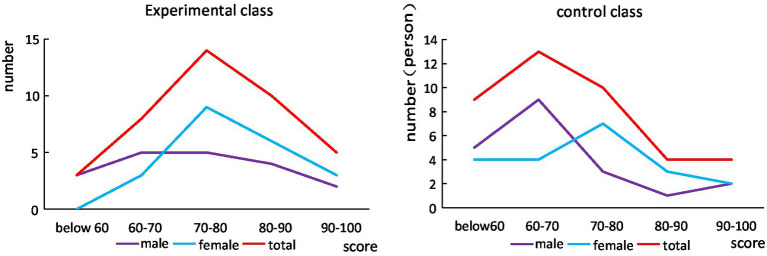
Comparison of the scores of the experimental class and the control class after the experiment.

It can be seen from Figure 8 that after a semester of human-computer interaction teaching, the overall academic performance of students has improved. The proportion of students in low grades has decreased significantly, and the excellent rate has increased significantly, but the proportion of students in high grades has not increased significantly, which shows that for high grade students, the effect of learning is not significant. Students in the control group improved their grades after one semester of study, but overall, the improvement was not high. Although the number of low-level students has decreased, there has been no significant increase in high-level students. In general, human-computer interaction teaching is more effective for students with low grades, while students with excellent grades already have their own learning methods, which generally does not improve them much.

**Figure 9:**
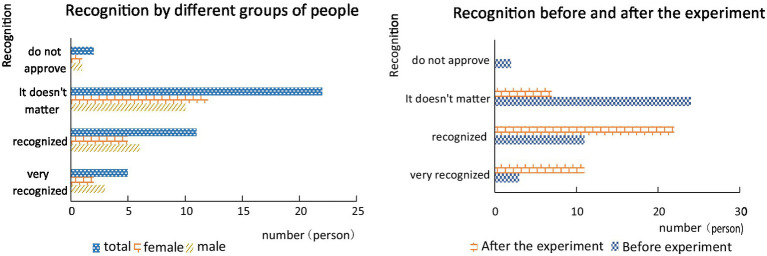
Research report on students’ recognition of interactive teaching.

As can be seen from Figure 9, before and after the experiment, students’ recognition of the human-computer interaction teaching mode has changed a lot. After the experiment, the students’ understanding of human-computer interaction was further deepened, and their recognition of the human-computer interaction model was also improved. There are also significant differences in gender recognition. Compared with girls, boys have a higher degree of recognition of human-computer interaction teaching, which may be the reason that human-computer interaction teaching has greatly improved boys’ performance.

**Figure 10:**
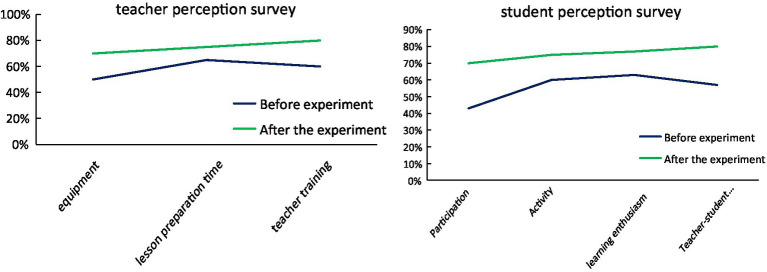
A survey of teachers and students’ perceptions of interactive teaching.

In Figure 10, teachers’ demands are more reflected in the improvement of current teaching, and they also point out their dilemma for human-computer interaction teaching. The school’s equipment cannot fully meet the students’ needs for human-computer interaction, and some senior teachers are unfamiliar with the human-computer interaction teaching mode and need further study and training. Students have a good response to the human-computer interaction model, which can better help students integrate into the class, integrate into their classmates, and learn English better.

## Discussion

In the above experiments, we tested the impact of human-computer interaction teaching on students’ English teaching. The experimental results confirmed the superiority of human-computer interaction teaching in improving students’ performance, suggesting that schools and teachers should further optimize teaching methods and try human-computer interaction teaching. This can further help students grow, create a good English learning environment, and help students improve their English learning ability. However, the experiment also pointed out that the current learning equipment has not fully pointed out the human-computer interaction, and the human-computer interaction requires more time and energy for teachers, and some teachers do not know enough about the human-computer interaction model, which restricts the further advancement of the human-computer interaction model. In the future, schools should increase investment in human-computer interaction equipment and at the same time pay attention to the training of teachers, improve their human-computer interaction teaching ability, and better help students master English smoothly.

## Conclusion

This paper conducts research on human-computer interactive English learning from the perspective of social cognition in the age of intelligence. It introduces social cognitive theory and human-computer interaction technology in detail, and verifies the impact of human-computer interaction teaching on current English teaching. Experiments have shown that human-computer interaction can better help students improve their English performance and enhance their interest and fun in English learning. This has a certain promotion significance for optimizing the English teaching mode and cultivating high-quality students, which can better help students grow. However, there are also certain problems in the experimental process: the sample selected in this paper is too small and may not be representative. The experimental time in this paper is not long and cannot represent the overall situation, which may cause certain errors in the experimental results. The indicators selected in this research may be too subjective, there are no quantitative indicators, and sometimes it is difficult to measure the representativeness of the indicators. Looking forward to further strengthening the advantages of human-computer interactive teaching in the future, while making up for the subjective indicators and small samples, expanding the scope of the pilot, we hope further efforts in these aspects will made to better students enjoy the fun of learning English.

## Author’s Note

QY was born in Nanchang, Jiangxi, P.R. China, in 1989. She received the master‘s degree from Jiangxi Normal University, Jiangxi P.R. China. Now, she works in College of Foreign Language, Jiangxi University of Technology. Her research interest includes cognitive linguistics and computational linguistics. E-mail: yql@jxut.edu.cn.

## Data Availability Statement

The original contributions presented in the study are included in the article/Supplementary Material, further inquiries can be directed to the corresponding author.

## Author Contributions

QY: work concept or design, the data collection, and draft paper.

## Funding

This work was supported by Jiangxi Provincial Social Science Foundation of China (grant no. 21YY32)—A Research of Chinese Humorous Diplomatic Discourse from the Perspective of Neo-Cognitive Pragmatics, by Jiangxi University of Technology Teaching Reform Foundation (grant no. JG1909)—A New Teaching Mode in the Flipped Classroom of “Society and Culture in English Countries” against the Background of “Internet +”, and by the Humanities and Social Sciences Research Fund of Jiangxi University of Technology (grant no. RW1913) - A Study on Conceptual Metaphors of “Moon” in Chinese Classical Poems.

## Conflict of Interest

The author declares that the research was conducted in the absence of any commercial or financial relationships that could be construed as a potential conflict of interest.

## Publisher’s Note

All claims expressed in this article are solely those of the authors and do not necessarily represent those of their affiliated organizations, or those of the publisher, the editors and the reviewers. Any product that may be evaluated in this article, or claim that may be made by its manufacturer, is not guaranteed or endorsed by the publisher.
